# The ancestral salicylic acid biosynthesis pathway in plants

**DOI:** 10.1016/j.tplants.2025.08.004

**Published:** 2026-01

**Authors:** Jie Huang, Renier A.L. van der Hoorn

**Affiliations:** 1 The Plant Chemetics Laboratory, Department of Biology, University of Oxford, Oxford, OX1 3RB, UK

**Keywords:** plant hormone, salicylic acid, phenylalanine ammonia-lyase pathway, SA biosynthesis

## Abstract

Salicylic acid (SA) is a vital phytohormone produced from isochorismate in arabidopsis (*Arabidopsis thaliana)*. However, SA in most plant species is produced from phenylalanine, a pathway that has long remained unresolved. Three recent studies filled this major knowledge gap and elucidated a multistep SA biosynthesis pathway that is ancestral in the plant kingdom.

## Main text

**Figure 1:**
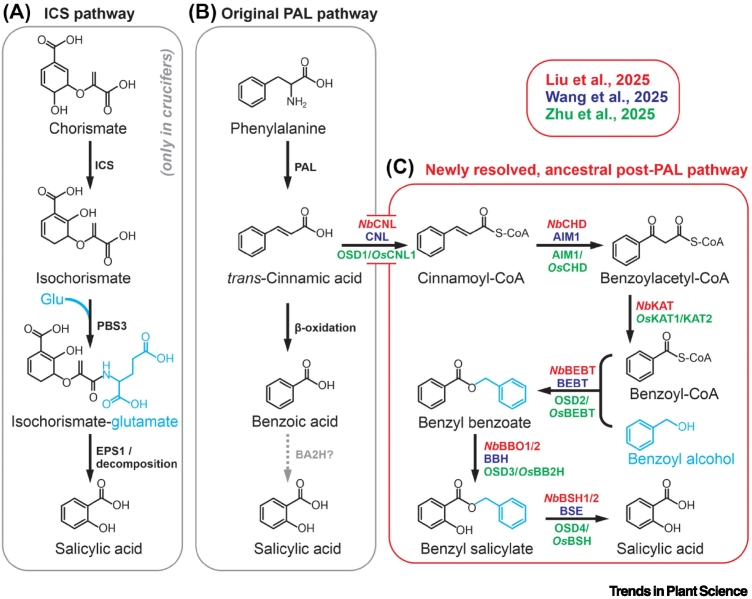
Salicylic acid (SA) biosynthesis in plants resolved. (A) SA biosynthesis through the isochorismate synthase (ICS) pathway. In arabidopsis (*Arabidopsis thaliana*) and other crucifers, SA is predominantly synthesised via the ICS pathway, where ICS converts chorismate into isochorismate, followed by a two-step transformation into SA mediated by PBS3 and EPS1 (or spontaneously decomposes). (B) Originally proposed SA biosynthesis through the phenylalanine ammonia-lyase (PAL) pathway. Phenylalanine is deaminated by PAL to yield *trans*-cinnamic acid, which is subsequently converted into benzoic acid (BA) through β-oxidation. Benzoic acid is then assumed to be hydroxylated by putative benzoic acid 2-hydroxylase (BA2H) to produce SA. (C) Recently resolved SA biosynthesis pathway [11,12,15]. Rice synthesizes benzoyl-CoA via a β-oxidative pathway involving OsPAL6, SA-DEFICIENT GENE (OSD)-1, AIM1, and OsKAT1/2. Benzoyl-CoA is then converted into SA via benzyl benzoate and benzyl salicylate (BS), mediated by OSD2, OSD3, and OSD4, which encode benzoyl-CoA:benzyl alcohol benzoyltransferase (BEBT), BB hydroxylase (BBH), and BS esterase (BSE), respectively. These findings highlight the sequential enzymatic actions of BEBT, BBH, and BSE in the SA biosynthetic pathway in rice. Likewise, in *Nicotiana benthamiana,* benzoyl-CoA and benzoyl alcohol are conjugated by BEBT to form benzyl benzoate, which is then hydroxylated by BBO to generate benzyl salicylate, which hydrolyses to release SA.

SA was first identified as a signaling molecule in tobacco disease resistance in 1979 [1]. Since then, extensive studies have demonstrated that SA has a vital role in plant defense responses to biotic and abiotic stresses. Moreover, exogenous application of SA and its analogs boosts immune responses in plants [2]. Despite its well-established function in plant immunity, SA biosynthesis is not fully understood. Two distinct pathways for SA biosynthesis have been proposed in plants. In the model plant arabidopsis (*Arabidopsis thaliana*), SA is primarily synthesized via the isochorismate synthase (ICS) pathway, in which Isochorismate Synthase-1 (ICS1) converts chorismate to isochorismate, followed by a two-step conversion to SA mediated by either PBS3 and EPS1, or by the latter via spontaneously decomposition [3., 4., 5.] (Figure 1A). However, orthologs of PBS3 and EPS1 are largely absent in species outside the crucifer plant family [6]. Moreover, genetic evidence from *ics* mutants suggests that ICS is not required for SA biosynthesis in rice [7], indicating that the ICS pathway is not the predominant route of SA biosynthesis in all plant species.

In addition to the ICS pathway, biochemical studies with isotope-labeled precursors indicated that SA can also be produced from phenylalanine via phenylalanine ammonia-lyase (PAL) [8]. In this pathway, phenylalanine undergoes deamination by PAL to generate *trans*-cinnamic acid (*t*-CA), which is subsequently converted to benzoic acid (BA) through β-oxidation [8,9]. BA is then thought to be hydroxylated by a putative benzoic acid 2-hydroxylase (BA2H) to yield SA. However, the existence of BA2H has never been shown directly [10] (Figure 1B), and the molecular mechanisms underlying this conversion remain poorly understood.

Three recent studies challenge the longstanding hypothesis that SA is derived from the hydroxylation of BA. Instead, their findings demonstrate that the critical hydroxylation step occurs at the level of benzyl benzoate (BB). To dissect the SA biosynthetic pathway, Zhu and colleagues [11] performed a high-throughput forward genetic screen for SA-deficient mutants from ethyl mesylate sulfonate (EMS)-mutagenized rice populations, and identified *Oryza sativa SA-DEFICIENT GENE (OSD1*, previously known as *CNL*). *OSD2, OSD3*, and *OSD4*, which are also involved in SA biosynthesis, were subsequently identified through reverse genetics. Integrating their genetic evidence with previous findings, the authors proposed that the PAL–SA pathway comprises two major modules: (i) the production of benzoyl-CoA from *t-CA* via β-oxidation; and (ii) the conversion of benzoyl-CoA into SA through a series of ligation, hydroxylation, and hydrolysis reactions.

Genetic and biochemical analyses confirmed that the β-oxidative route to benzoyl-CoA biosynthesis in rice is mediated sequentially by OSD1, AIM1, and OsKAT1/OsKAT2 (Figure 1C). Furthermore, they also demonstrated that SA is produced from benzoyl-CoA via the intermediates BB and benzyl salicylate (BS), through the sequential actions of rice OSD2, OSD3, and OSD4 (Figure 1C). Further bioinformatic analysis indicated that the PAL–SA pathway evolved in a stepwise manner throughout plant evolution, with the fully functional pathway established before the divergence of gymnosperms. It has since been conserved in seed plants, although specific components, such as OSD3 and OSD4, have been lost in crucifers, including arabidopsis. This study uncovers a novel PAL–SA biosynthetic pathway in rice, providing a mechanistic framework for understanding SA production in monocots.

Similarly, Wang and colleagues [12] used a reverse genetic strategy to elucidate the biosynthetic pathway of SA in rice. Since benzoyl-CoA is large in terms of its molecular size, the authors hypothesized that the benzoyl-CoA-converting enzyme resides within peroxisomes. Given that CNL has a critical role in SA biosynthesis during rice seed germination under water submergence [13] and CNL also exhibits a more specific role in SA biosynthesis compared with PAL or AIM1 [14], they hypothesized that downstream pathway components would be co-expressed with *CNL*. This analysis revealed a strong co-expression network linking *CNL* with a gene encoding benzoyl-CoA:benzyl alcohol benzoyltransferase (*BEBT*), which colocalizes with CNL and β-oxidation enzymes in peroxisomes. Metabolite analysis on *bebt* mutants, particularly those involving stable isotope-labeled BA feeding, rejected the previous BA2H model and established BB as a key intermediate. Subsequently, they identified BB hydroxylase (BBH) and BS esterase (BSE) using co-expression analysis followed by biochemical and genetic characterization. They then confirmed that these three enzymes sequentially convert benzoyl-CoA into BB, then into BS, and finally into SA. Furthermore, they established the importance of this BEBT–BBH–BSE module for pathogen-induced SA production in several key crop species, including wheat, tomato, and cotton. Comparative gene expression analyses also indicated that, in diverse species outside the Brassicaceae, this PAL pathway represented by the BEBT–BBH–BSE module, is more prominently induced by pathogens compared with the ICS pathway. These findings highlight that both the SA biosynthetic function and the pathogen-induced expression of this three-enzyme module are conserved across many plant species.

Moreover, Liu *et al.* [15] elucidated three key enzymatic steps involved in SA biosynthesis in *Nicotiana benthamiana*. The authors silenced several *PAL* genes and two *ICS* genes that are transcriptionally changed upon infection with SA-inducing *Pseudomonas syringae* pv. *tomato* DC3000 and demonstrated that SA is predominantly synthesised via the PAL pathway in this species. To uncover additional components required for SA biosynthesis, Liu *et al.* performed a forward genetic screen and discovered that a gene encoding BEBT is essential for SA accumulation following pathogen infection [15]. Functional characterization revealed that *Nb*BEBT is essential for BB biosynthesis in *N. benthamiana*. This intermediate is subsequently hydroxylated by two cytochrome P450s, *Nb*BBO1 and *Nb*BBO2, to form BS, which is then hydrolyzed by *Nb*BSH1 and *Nb*BSH2 to release SA (Figure 1C). Orthologs of these enzymes are conserved across most seed plants, except in some crucifers, such as arabidopsis, *Brassica*, and *Raphanus*, which have evolved an alternative SA biosynthesis via ICS. Moreover, genes from willow, poplar, soybean, and rice can complement SA-deficient *N. benthamiana* mutants, indicating that this pathway is functionally conserved in both dicots and monocots. Furthermore, Liu *et al.* also demonstrated the roles of *Os*BEBT, *Os*BBO, and *Os*BSH in SA biosynthesis using CRISPR-knockout mutants in rice [15]. These findings uncover a widely conserved and distinct route for SA production in plants.

Unlike the ICS pathway, which is predominant in crucifers, the newly identified PAL–SA biosynthesis pathway is conserved across seed plants, including crop species, such as rice, wheat, tomato, cotton, and soybean. These three studies significantly advance our understanding of the diversity and evolution of SA biosynthetic routes in the plant kingdom and open new avenues to investigate regulatory mechanisms of SA production in a range of species beyond the crucifers. Elucidating the functional contribution of this new pathway to plant immunity across diverse plant species is critical for exploiting its potential in crop protection strategies. Zhu *et al.* [11] demonstrated that rice maintains high basal levels of SA independently of pathogen infection, likely through spatial regulation and potential metabolic channeling between PAL–SA biosynthetic enzymes. While overexpressing downstream enzymes did not boost SA levels, activation of the pathway via OSD1 significantly enhanced SA accumulation and disease resistance. Further supporting this, transient expression assays in *N. benthamiana* performed by Wang *et al.* [12] revealed that only CNL (OSD1) expression significantly increased SA levels, whereas individual expression of BEBT, BBH, or BSE had minimal effect. Notably, co-expressing CNL with BEBT further increased SA production, surpassing levels achieved by CNL alone. These findings highlight OSD1/CNL as a promising target for engineering durable immunity in plants. Overall, the three studies provide a foundational framework for extending SA biosynthesis research beyond the traditional arabidopsis model, offering valuable insights for both fundamental plant biology and the development of sustainable agricultural strategies.

## Declaration of interests

None declared by authors.

## References

[1] White R.F. (1979). Acetylsalicylic acid (aspirin) induces resistance to tobacco mosaic virus in tobacco. Virology.

[2] Lefevere H. (2020). Salicylic acid biosynthesis in plants. Front. Plant Sci..

[3] Wildermuth M.C. (2001). Isochorismate synthase is required to synthesize salicylic acid for plant defence. Nature.

[4] Torrens-Spence M.P. (2019). PBS3 and EPS1 complete salicylic acid biosynthesis from isochorismate in *Arabidopsis*. Mol. Plant.

[5] Rekhter D. (2019). Isochorismate-derived biosynthesis of the plant stress hormone salicylic acid. Science.

[6] Hong K. (2025). Emergence of isochorismate-based salicylic acid biosynthesis within Brassicales. Proc. Natl. Acad. Sci. U. S. A..

[7] Wang Z. (2024). Isochorismate synthase is required for phylloquinone, but not salicylic acid biosynthesis in rice. aBIOTECH.

[8] Yalpani N. (1993). Pathway of salicylic acid biosynthesis in healthy and virus-inoculated tobacco. Plant Physiol..

[9] Bussell J.D. (2014). Peroxisomal ATP-binding cassette transporter COMATOSE and the multifunctional protein abnormal INFLORESCENCE MERISTEM are required for the production of benzoylated metabolites in *Arabidopsis* seeds. Plant Physiol..

[10] Sawada H. (2006). Induction of benzoic acid 2-hydroxylase and salicylic acid biosynthesis–Modulation by salt stress in rice seedlings. Plant Sci..

[11] Zhu B. (2025). Complete biosynthesis of salicylic acid from phenylalanine in plants. Nature.

[12] Wang Y. (2025). Deciphering phenylalanine-derived salicylic acid biosynthesis in plants. Nature.

[13] Wang Y. (2024). A peroxisomal cinnamate:CoA ligase-dependent phytohormone metabolic cascade in submerged rice germination. Dev. Cell.

[14] Wang Y. (2024). Species- and organ-specific contribution of peroxisomal cinnamate:CoA ligases to benzoic and salicylic acid biosynthesis. Plant Cell.

[15] Liu Y. (2025). Three-step biosynthesis of salicylic acid from benzoyl-CoA in plants. Nature.

